# Modeling Relapsing Disease Dynamics in a Host-Vector Community

**DOI:** 10.1371/journal.pntd.0004428

**Published:** 2016-02-24

**Authors:** Tammi L. Johnson, Erin L. Landguth, Emily F. Stone

**Affiliations:** 1 Division of Biological Sciences, University of Montana, Missoula, Montana, United States of America; 2 Department of Mathematical Sciences, University of Montana, Missoula, Montana, United States of America; UC Davis School of Medicine, UNITED STATES

## Abstract

Vector-borne diseases represent a threat to human and wildlife populations and mathematical models provide a means to understand and control epidemics involved in complex host-vector systems. The disease model studied here is a host-vector system with a relapsing class of host individuals, used to investigate tick-borne relapsing fever (TBRF). Equilibrium analysis is performed for models with increasing numbers of relapses and multiple hosts and the disease reproduction number, *R*_*0*_, is generalized to establish relationships with parameters that would result in the elimination of the disease. We show that host relapses in a single competent host-vector system is needed to maintain an endemic state. We show that the addition of an incompetent second host with no relapses increases the number of relapses needed for maintaining the pathogen in the first competent host system. Further, coupling of the system with hosts of differing competencies will always reduce *R*_*0*_, making it more difficult for the system to reach an endemic state.

## Author Summary

An important development in the study of infectious diseases is the application of mathematical models to understand the interplay between various factors that determine epidemiological processes. Vector-borne diseases are additionally complex with interactions between multiple host and vector species. Understanding the transmission dynamics of vector-borne diseases is an important step towards controlling outbreaks and mitigating human infection risk. Identifying the biotic and abiotic interactions and mechanisms that may contribute to disease emergence, establishment and persistence is necessary for assessing current and future disease risk, as well as developing effective control strategies. Tick-borne relapsing fever (TBRF) is found around the world and is caused by several species of *Borrelia* spirochetes, which are vectored by soft ticks of the genus *Ornithodoros*. TBRF is a cryptic disease that still causes significant morbidity and mortality, especially in some African countries. Here, we develop and adapt a compartmentalized mathematical model (SIR) with a relapsing component to investigate the dynamics of TBRF.

## Introduction

An important development in the study of infectious diseases is the application of mathematical models to understand the interplay between various factors that determine epidemiological processes. Many systems show a rich variety of dynamics that arise from nonlinear interactions (due to the mixing of different infectious populations) or temporal forcing (caused by changes in the average contact rate) [1]. Vector-borne diseases are additionally complex with interactions between multiple host and vector species [2–4]. Compartmental models, such as susceptible, infectious, and removed models (SIR) [5], have been applied to many disease systems in an effort to examine system dynamics. In these epidemic models, susceptible individuals pass into the infective class, from which they transition to the removed class. For some diseases, recovered individuals may relapse with a reactivation of infection and revert back to an infective class. An example of such a system is found in van den Driessche et al. [6], which included a relapsing rate between the susceptible and the same infected compartment. Adding additional infected compartments simulates disease systems in which there is a relapsing component, leading to a prolonged infectious period, presumed to be important to disease persistence. To our knowledge, the addition of a relapsing component has not been applied to a host-vector system. Noteworthy vector-borne relapsing diseases include tick-borne relapsing fever (TBRF) and malaria.

An advantage of these types of models is the ability to vary parameters, while monitoring the overall effect on the disease system, allowing for the exploration of characteristics of the system that may not be well understood. Tick-borne relapsing fever (TBRF) is a cryptic disease that still causes significant morbidity and mortality worldwide, especially in African countries [7–10]. TBRF is a vector-borne zoonotic disease endemic to central Asia, Africa, and the Americas [11], and is caused by infection with *Borrelia* spirochetes. All but one species of relapsing fever spirochetes are vectored by soft ticks (*Ornithodoros* spp.) [12]. Relapsing fever is characterized by recurring febrile episodes and generalized symptoms including headache, chills, myalgia, nausea, and vomiting[13]. There is a rapid onset of disease symptoms, with a febrile episode lasting 3–6 days, after which symptoms subside, only to return in 7–10 days. Symptoms are associated with large number of spirochetes present in the bloodstream (spirochetemia), and subside when the host generates an antibody response against the variable major proteins (Vmps). The Vmps are involved with antigenic variation, and relapsing fever Borrelia produce a new variant during infection, subsequently attaining high densities [14, 15]. Little is known regarding the number of relapses in natural hosts, but studies have shown a range from 1 to 5 in experimentally infected animals [16]. In humans, there is an average relapse rate of three febrile episodes without treatment, but up to 13 relapses have been observed [17].

*Ornithodoros* spp. ticks are long-lived, fast feeding vectors that are known to live > 10 years, and have been shown to survive for up to five years without feeding [18]. *Ornithodoros* ticks are nidicolous ticks that rarely leave the confines of the host nest or burrow and are able to obtain a blood meal and detach from the host in < 90 min. Additionally, soft ticks only obtain a blood meal about once every 3 months; even when presented with the opportunity to feed daily. *Ornithodoros* ticks require several months between feedings and can survive years between feeding. The longevity of these ticks means that they outlive their rodent hosts, affording the potential to infect several cohorts of rodents over the course of the tick lifespan. Once infected ticks remain infected and infectious for the duration of their lifespan.

Here, we model TBRF caused by infection with *B*. *hermsii* and vectored by *O*. *hermsi*. We parameterize the model with field-derived values from hosts on Wild Horse Island in Montana and a single genomic group I (GGI) strain of *B*. *hermsii*. The overall goal of this study was to develop a SIR model using TBRF dynamics to describe a host-vector system with a relapsing class of host individuals. First, using specific information from a TBRF system located on Wild Horse Island, MT, a model for the dynamics of a single host-vector interaction was developed. For models with increasing numbers of relapses and multiple hosts, equilibrium analysis was performed and *R*_*0*_ was generalized. Parameter values were considered in the model to provide theoretical criteria for population stability and to determine the parameters that would result in elimination of the disease. Finally, single and coupled host-vector systems were explored, focusing on the addition of less competent hosts and the number of relapses needed in order to maintain an endemic equilibrium. We use the model to ask several important biological questions pertaining to the TBRF system determining effect adding relapsing classes has on pathogen persistence and the effect of multiple host species with varying competency for acquiring and transmitting *B*. *hermsii*.

## Methods

### Study system

We sought to develop a model based on disease dynamics on Wild Horse Island (WHI), Flathead Lake, Lake County, MT. WHI is the largest island (~2100 acres) on Flathead Lake and like other islands on the lake has a limited diversity of rodent host species. WHI is almost exclusively inhabited by deer mice (*Peromyscus maniculatus*) and pine squirrels (*Tamiasciurus hudsonicus*) as the terrestrial rodents and provided an important opportunity to develop and parameterize a model including only two hosts. Although there are two genomic groups (GGI and GGII) of *B*. *hermsii* present on WHI, we parameterize the model using estimates for only GGI *B*. *hermsii*, as host competency experiments have primarily been performed with GGI *B*. *hermsii* [16].

### Dynamical systems model

A key assumption for host-vector disease modeling is the definition of the transmission term, which represents the contact between hosts and vectors. The formulation of the transmission term affects the reproduction number, *R*_*0*_, which is a central predictor of disease systems [19]. For host-vector disease models, the transmission term includes the vector biting rate. This rate controls the pathogen transmission both from the vector-to-host and from the host-to-vector. The TBRF model follows frequency-dependent transmission assumptions through the biting rate, since a blood meal is only required approximately once every three months regardless of the host population density. Following this framework, hosts would likely experience an increasing number of bites as the vector population increased.

Given a mathematical model for disease spread, *R*_*0*_ is an essential summary parameter. It is defined as the average number of secondary infections produced when one infected individual is introduced into a completely susceptible host population [20]. When *R*_*0*_ < 1, the disease free equilibrium (DFE) at which the population remains in the absence of disease is locally asymptotically stable. However, if *R*_*0*_ > 1, then the DFE is unstable and invasion is always possible (see [21]) and a new endemic equilibrium (EE) exists. For this study, *R*_*0*_ was extracted following the methodology developed in van den Driessche et al. [22] (see also [23, 24]) for general compartmental disease models, which can be extended to more complicated host-vector disease systems [25, 26].

### Parameter estimates

Specific parameter values for this system have not yet been determined, but can be estimated from similar studies and from data collected on *O*. *hermsi* from laboratory experiments. The units of the rates are individuals per month. Table 1 summarizes the notation for all system parameters and variables. See Table 2 for specific model values used in all of the host-vector models. Note that parameters denoted with additional subscripts of *ps* and *dm* refers to values specific to the pine squirrel and deer mouse host-vector systems, respectively.

**Table 1:** Parameters and variable notation in the host-vector TBRF model for *j—*1 relapses between *j* infected compartments (rates are per month, competency values are probabilities (per bite)) and dimensionless forms (rescaled by γ or normalized by *N*(0)). In the coupled system, additional subscripts with *ps* represent the pine squirrel host-vector system and *dm* represents the deer mouse host-vector system.

Notation	Description	Dimensionless
*S*	Host susceptible	*s = S / N*(0)
*I*_*j*_	Host infected from infected population *j*	*i*_*j*_ *= I*_*j*_ */ N*(0)
*R*	Host removed	*r = R / N*(0)
*N*	Host total	*n = N / N*(0)
*c*	Host competency	*l = fc / γ*
*γ*	Host recovery rate	*1*
*α*_*j-1*_	Host relapse rate for *j* infected compartments	*q*_*j-1*_ *= α*_*j-1*_ */ γ*
*β*	Host growth rate	*a = β / γ*
*μ*	Host death rates	*b = μ/ γ*
*S*_*v*_	Vector susceptible	*s*_*v*_ *= S*_*v*_ */ N*(0)
*I*_*v*_	Vector infected	*i*_*v*_ *= S*_*v*_ */ N*(0)
*N*_*v*_	Vector total	*n*_*v*_ *= N*_*v*_ */ N*(0)
*c*_*v*_	Vector competency	*k = fc*_*v*_ */ γ*
*β*_*v*_	Vector growth rate	*a*_*v*_ *= β*_*v*_ */ γ*
*μ*_*v*_	Vector death rates	*b*_*v*_ *= μ*_*v*_ */ γ*
*f*	Biting rate between vector-host system	*l = fc / γ; k = fc*_*v*_ */ γ*

**Table 2:** Parameter values in the TBRF model for *j-1* relapses between *j* infected compartments (rates are per month, competency values are probabilities per bite). The subscripts *ps* and *dm* denote values used in the pine squirrel and deer mouse host-vector system, respectively. Note that if the subscripts do not appear, then the parameter is the same value in both systems.

Notation	Description	Value
*S*_*ps*_(0), *S*_*dm*_(0)	Initial host susceptible	850, 10,000
*I*_*j*,*ps*_(0), *I*_*j*,*dm*_(0)	Initial host infected from infected population *j*	0
*R*_*ps*_(0), *R*_*dm*_(0)	Initial host removed	0
*N*_*ps*_(0), *N*_*dm*_(0)	Initial host total	850
*c*_*ps*_	Pine squirrel host competency	0.9
*c*_*dm*_	Deer mouse host competency (coupled system)	0.2
*γ*, α_1,….,_ α_j-1_	Host transition rates	4.35
*β*_ps_, *β*_,dm_	Host growth rate	0.33, 1.0
*μ*_s,ps_, *μ*_s,dm_	Host susceptible death rate	0.33/(*j*+2), 1.0/(*j*+2)
*μ*_ij,ps_, *μ*_ij,dm_	Host infected death rate from infected population *j*	0.33/(*j*+2), 1.0/(*j*+2)
*μ*_r,ps_, *μ*_r,dm_	Host removed death rate	0.33/(*j*+2), 1.0/(*j*+2)
*S*_*v*,*ps*_(0), *S*_*v*,*dm*_(0)	Initial vector susceptible	9,900
*I*_*v*,*ps*_(0), *I*_*v*,*dm*_(0)	Initial vector infected	100
*N*_*v*,*ps*_(0), *N*_*v*,*dm*_(0)	Initial vector total	10,000
*c*_*v*_	Vector competency	0.95
*β*_*v*_	Vector growth rate	2.08
μ_sv_ = μ_iv_	Vector death rates	1.04
*F*	Vector biting rate	0.33

The birth rates for host and vector are each set to a constant value (*β* and *β*_*v*_, respectively) and the compartmental death rates (for host and vector) are identical and set equal to birth rate. Then the death rates must be

$$\mu_{\text{s}}=\mu_{\text{i}1}=\cdots =\mu_{\text{ij}}=\mu_{\text{r}}=\frac{\beta }{\text{j}+2}$$ (1)

and

$$\mu_{sv}=\mu_{iv}=\frac{\beta_{v}}{2}.$$ (2)

The growth rate of pine squirrels (*β*_*ps*_ = 0.33 individuals per month) is an average of the rates found in the literature, i.e., four individuals per litter at 1 litter per year [27]. The growth rate of deer mice is also taken from average estimates from the literature; we estimate growth rate based on an average of three litters per year and four young per litter, (*β*_dm_ = 1 individual per month) [28]. The death rates are determined from Eq (1), which depends on the number of relapses in the system. For example, for a pine squirrel host-vector system with one relapse, all death rates would be 0.0825. Life history dynamics of *O*. *hermsi* are not well documented and virtually nothing is known about the reproductive behavior and survival of these ticks in nature. Conservative estimates from the laboratory show that soft-bodied ticks lay on average five clutches over their approximately 10 year lifespan with roughly 50 eggs per clutch [29] (T. Schwan *personal communication*). Thus, the vector birth rate is *β*_*v*_ = 2.08 individuals per month. Following Eq (2), we get death rates of *μ*_*sv*_ = *μ*_*iv*_ = 1.04 for the vector compartments.

The rate at which an individual transitions among infected compartments and to the removed compartment is fixed and is assumed to be the same for all compartments. As more infected compartments are added to the system, the corresponding constant rates are *γ* = *α* = *α*_*1*_ = … = *α*_*j-1*_, for *j* infected compartments. Field parameter estimates have not yet been made for these transition rates (i.e., relapse and recovery rates). Laboratory results from three pine squirrels indicate a transition rate of approximately 4.35 individuals per month for a single compartment (Burgdorfer and Mavros 1970). Then *γ* = *α* = *α*_*1*_ = … = *α*_*j-1*_ = 4.35.

Ticks are assumed to bite a host once every three months (i.e., *f* = 0.33). Competency values are between 0 and 1 and thus modify the transmission rate of the infection by multiplying the biting rate. Burgdorfer and Mavros [16] observed a high competency in pine squirrels successfully infecting 3/3 animals by tick bite or injecting them with triturated ticks. Using the same methods, they challenged deer mice with *B*. *hermsii* and were unsuccessful in establishing infection. Thus, we used competency values *c*_*v*_ = 0.95 for the probability of transmission for vectors, *c*_*ps*_ = 0.90 for pine squirrels, and *c*_*dm*_ = 0.10 for deer mice.

The carrying capacity for the pine squirrel and deer mouse system is determined specifically for WHI. On WHI there are approximately 425 ha of suitable habitat for pine squirrels with up to a maximum of 2 individuals per suitable habitat patch and approximately 850 ha of suitable deer mouse habitat with a conservative estimate of just less than 12 mice per ha [28]. Thus, the total number of pine squirrels (*N*_*ps*_) is estimated at 850 and total number of deer mice (*N*_*dm*_) is estimated at 10,000. The soft bodied tick population (*N*_*v*_) is virtually unknown, however, we assume that they are limited to the nests of their hosts. Initial field collections have found as many as 14 ticks in one nest on the island [30]; other collection efforts show > 300 ticks can be collected from a single nest or snag [31]. Because the estimates of ticks per nest vary largely between our limited collection on WHI and the literature we chose a conservative number of ticks. We estimate that each squirrel has less than one nest (because of juveniles in the system), and each nest is inhabited by 14 ticks. We found no ticks in nest material collected from deer mice, however, nest material collected during the human outbreak in 2002 yielded 14 *O*. *hermsi*; the carcasses of two deer mice were found nearby and American Robins (*Turdus migratorius*) had been nesting there [30]. Thus it is nearly impossible to estimate the average number of ticks in a deer mouse nest, or if in fact they are coming in contact with ticks while visiting other nests. We used an estimate of 20,000 total ticks on the island split equally among host systems. We chose a conservative estimate of 1% of all ticks are infected, as none of 12 of 14 field collected ticks were found to be infected [30]. Thus, we used *S*_*v*_(0) = 9,900 ticks for the single host-vector system and *S*_*v*_(0) = 19,800 ticks for the coupled host-vector system.

## Results

### Single host-vector system

**Fig 1:**
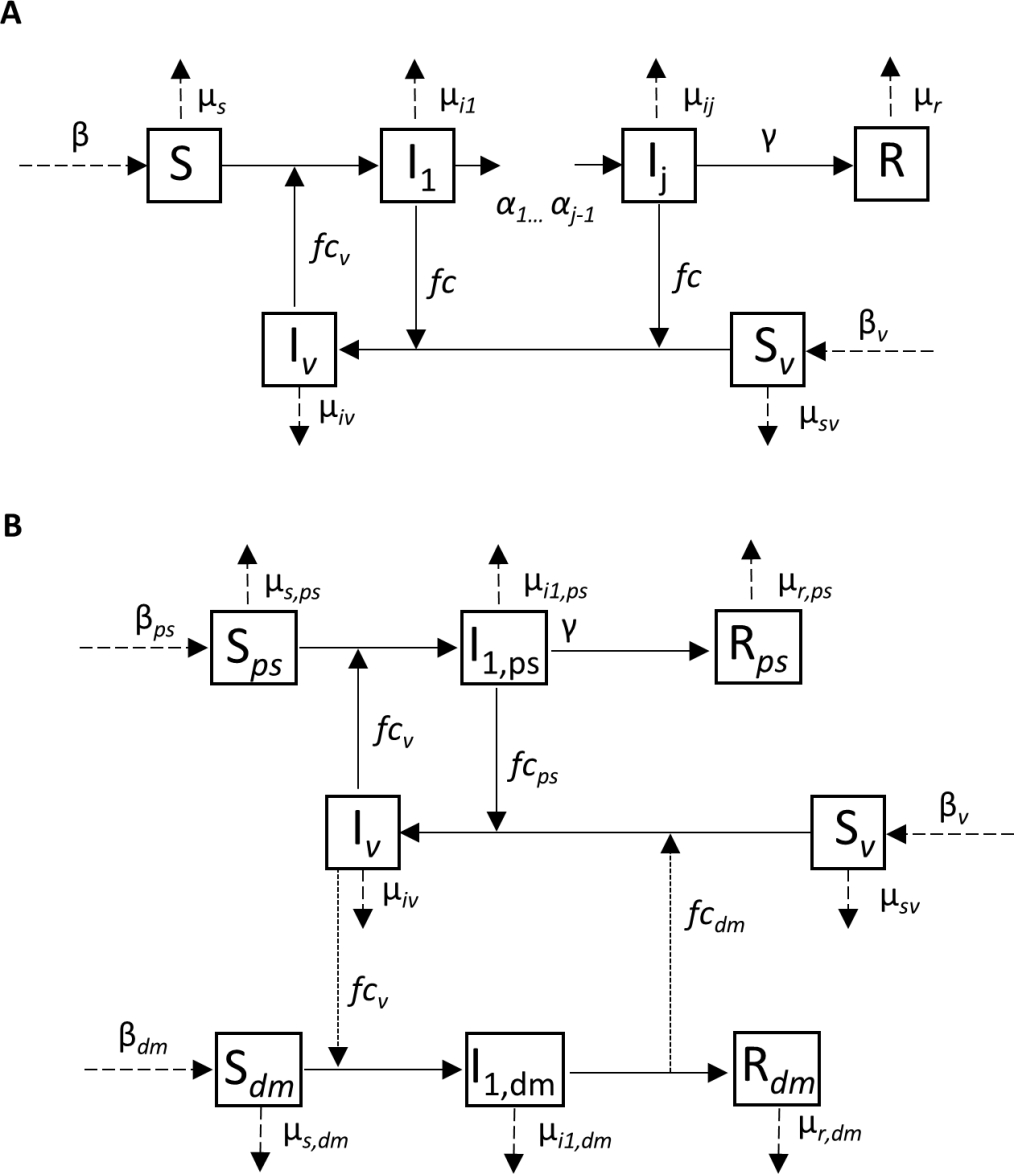
Conceptual models for the cross-infection dynamics between (a) a single host-vector system, which includes *j—*1 relapses between *j* infected compartments and (b) a coupled host-vector system with no relapses in either host. Dashed lines are vital rates for each population, where solid lines refer to interaction rates between compartments. See Table 1 for a summary of notation.

A model for the dynamics of TBRF in a single host-vector system is considered (see Fig 1A). The following assumptions are used to establish a model that is appropriate for the WHI TBRF system for the host pine squirrel and soft tick vector, *O*. *hermsi*. (1) The only sources of infection occur between the bite of an infective vector and susceptible host and between a bite of a susceptible vector and infective host (i.e. there are no horizontal or vertical transmission events). (2) The vector becomes infected and infectious for life immediately upon biting an infectious host. (3) The transmission terms are frequency-dependent through the biting rate, *f*. (4) The hosts relapse to different infected compartments (i.e. different serotypes within the hosts caused by antigenic variation) at rate *α* and recover from the disease at rate *γ*. (5) Though mortality rates are noted to differ for each compartment, we assume a constant total population for both hosts and vectors (*N* and *N*_*v*_, respectively). Thus, recruitment (or birth) and the sum of the removal (or death) rates from each compartment must be equal (Eqs 1 and 2).

The generalized system for the infection dynamics in a single host-vector system with *j*—1 relapsing rates for *j =* 1 infected compartments describes the number of susceptible hosts *S*(t), infectious hosts *I*_*k*_(t), removed hosts *R*(t), susceptible vectors *S*_*v*_(t), and infected vectors *I*_*v*_(t), where the total host population is $N=S+\sum_{k=1}^{j}I_{k}+R$ and the total vector population is *N*_*v*_ = *S*_*v*_ + *I*_*v*_ (see Fig 1A for a compartmental diagram and Table 1 for parameter definitions). The equations are

Host equations:

$$\begin{array}{c} {\overset{•}{S}}_{}=\beta S-fc_{v}I_{v}\frac{S}{N}-\mu_{s}S\\ {\overset{•}{I}}_{1}=fc_{v}I_{v}\frac{S}{N}-\alpha_{1}I_{1}-\mu_{i1}I_{1}\\ {\overset{•}{I}}_{2}=\alpha_{1}I_{1}-\alpha_{2}I_{2}-\mu_{i2}I_{2}\\ .\\ .\\ .\\ {\overset{•}{I}}_{j-1}=\alpha_{j-2}I_{j-2}-\alpha_{j-1}I_{j-1}-\mu_{i\left(j-1\right)}I_{j-1}\\ {\overset{•}{I}}_{j}=\alpha_{j-1}I_{j-1}-\gamma_{j}I_{j}-\mu_{ij}I_{j}\\ {\overset{•}{R}}_{j}=\gamma_{j}I_{j}-\mu_{r}R. \end{array}$$ (3)

Vector equations:

$$\begin{array}{c} {\overset{•}{S}}_{v}=\beta_{v}S_{v}-\frac{fcS_{v}}{N}\sum_{i=1}^{j}I_{i}-\mu_{sv}S_{v}\\ {\overset{•}{I}}_{v}=\frac{fcS_{v}}{N}\sum_{i=1}^{j}I_{i}-\mu_{iv}I_{v}. \end{array}$$ (4)

To evaluate the invasiveness of the disease in this system, we extracted *R*_*0*_ following the techniques developed by van den Driessche and Watmough [22] by sequentially adding infected compartments (see S1 for equilibrium analysis and derivations). The form of *R*_*0*_ was then inferred for *j—1* relapsing rates between *j* infected compartments as

$$R_{0}=f\sqrt{\frac{cc_{v}}{\mu_{iv}}\frac{S_{v}\left(0\right)}{N\left(0\right)}\left[\frac{1}{\alpha_{1}+\mu_{i1}}\left[1+\frac{\alpha_{1}}{\alpha_{2}+\mu_{i2}}\left[\cdots \left[1+\frac{\alpha_{j-1}}{\gamma +\mu_{ij}}\right]\right]\right]\right]}.$$ (5)

**Fig 2:**
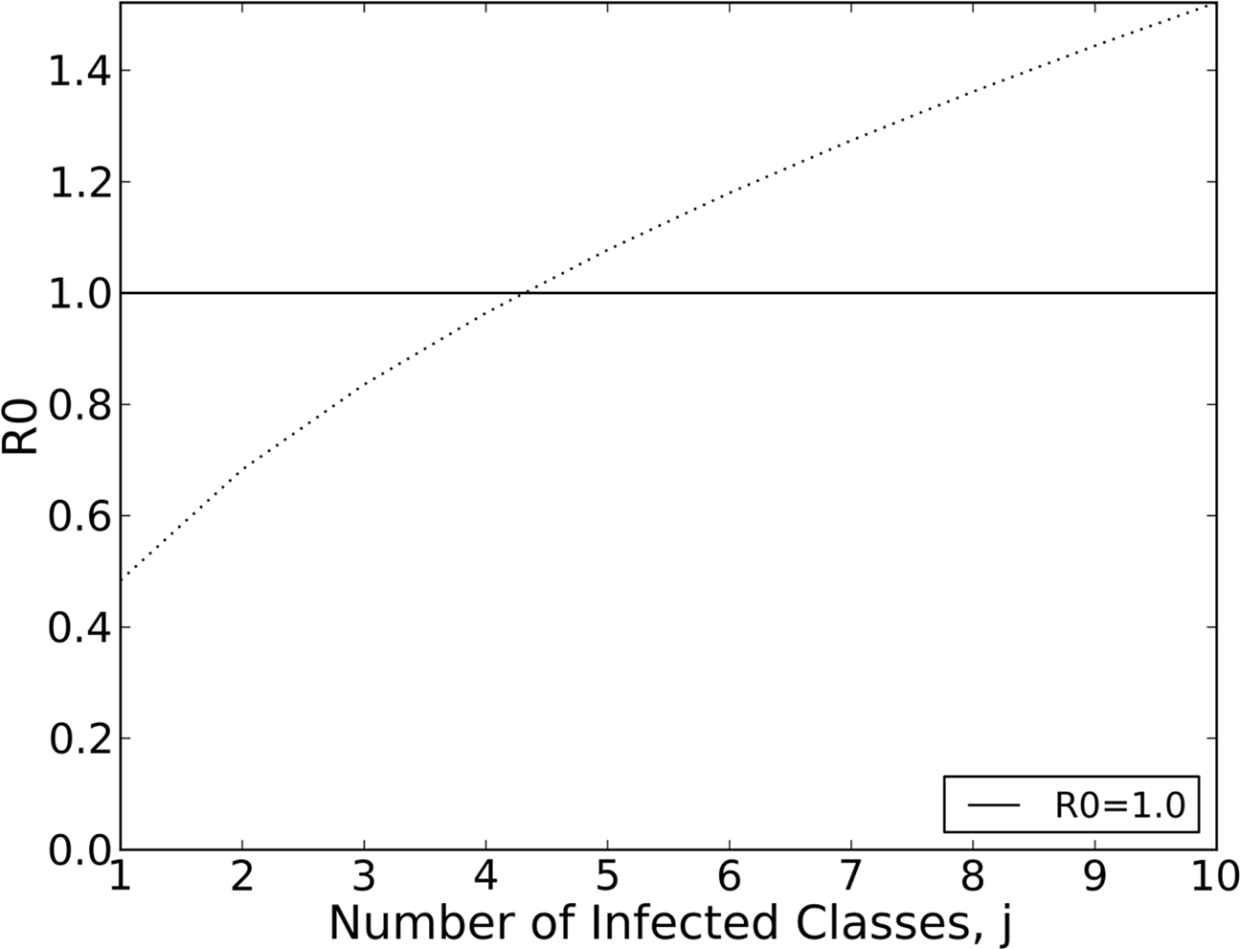
Single host-vector system. Increasing number of infected compartments are added to the single host-vector system and *R*_*0*_ is plotted (Eq 5). *R*_*0*_ becomes greater than one at four relapses.

*R*_*0*_ is directly proportional to the biting rate (*f*), competency values (*c* and *c*_*v*_), and the ratio of initial vectors to initial hosts $\left(\frac{S_{v}(0)}{N(0)}\right)$ and inversely proportional to the vector death rate (*μ*_*iv*_) and the rate that moves individuals out of the infected compartments (*α α*_1,….,_ α_j-1_, *μ*_*i1*_, …, *μ*_*ij*_, and *γ*). In addition, a pattern emerges as more infected compartments are added: a nesting sequence of terms that increase the value of *R*_*0*_ and potentially contribute to a change in stability of the DFE. To illustrate this concept, we used the pine squirrel host parameters (Table 2) for increasing number of infected compartments and plotted *R*_*0*_. *R*_*0*_ crosses 1 at between *j* = 4 and *j* = 5 infected compartments (i.e., four relapses; Fig 2).

### Coupled host-vector system

Here, the single host-vector model is expanded to include two hosts, namely pine squirrels and deer mice. Fig 1B is a compartmental diagram for the two systems with no relapses. The first host-vector system (*S*_*ps*_, *I*_*1*,*ps*_, *R*_*rs*_, *S*_*v*,*ps*_, *I*_*v*,*ps*_) is coupled with the second system (*S*_*dm*_, *I*_*1*,*dm*_, *R*_*dm*_, *S*_*v*,*dm*_, *I*_*v*,*dm*_) through ticks biting either host species, with parameter *f*, and is further controlled by competency values of either the ticks (*c*_*v*_) or hosts (*c*_*ps*_ or *c*_*dm*_ for pine squirrel and deer mice, respectively). Transmission occurs through three mechanisms: 1) *fc*_*v*_, which is the biting rate modified by the tick competency through which an infected tick bites a host from each system, 2) *fc*_*ps*_, which is the biting rate modified by the pine squirrel competency in that a susceptible tick bites an infected pine squirrel, and 3) *fc*_*dm*_, which is the biting rate modified by the deer mouse competency, such that a susceptible tick bites an infected deer mouse. The parameters remain as in the single host vector system, denoted with additional subscripts to represent the respective host-vector system (either *ps* or *dm*), and are explained in Tables 1–2.

The generalized system for the infection dynamics in a coupled host-vector system with *j*—1 relapsing rates for *j =* 1 infected compartments describes the pine squirrel system with the number of susceptible hosts *S*_*ps*_(t), infectious hosts *I*_*k*,*ps*_(t), and removed hosts *R*_*ps*_(t). The total pine squirrel host population is $N_{ps}=S_{ps}+\sum_{k=1}^{j}I_{k,ps}+R_{ps}.$ Likewise, the deer mouse host system consists of susceptible hosts *S*_*dm*_(t), infectious hosts *I*_*k*,*dm*_(t), and removed hosts *R*_*dm*_(t) with a total deer mouse host population of $N_{dm}=S_{dm}+\sum_{k=1}^{j}I_{k,dm}+R_{dm}.$ The vector compartments are susceptible vectors *S*_*v*_(t), infected vectors *I*_*v*_(t) and a total vector population of *N*_*v*_ = *S*_*v*_ + *I*_*v*_. The equations are

Pine squirrel host system:

$$\begin{array}{c} {\overset{•}{S}}_{ps}=\beta S_{ps}-fc_{v}I_{v}\frac{S_{ps}}{N_{ps}}-\mu_{s,ps}S_{ps}\\ {\overset{•}{I}}_{1,ps}=fc_{v}I_{v}\frac{S_{ps}}{N_{ps}}-\alpha_{1,ps}I_{1,ps}-\mu_{i1,ps}I_{1,ps}\\ {\overset{•}{I}}_{2,ps}=\alpha_{1,ps}I_{1,ps}-\alpha_{2,ps}I_{2,ps}-\mu_{i2,ps}I_{2,ps}\\ \vdots \\ {\overset{•}{I}}_{j-1,ps}=\alpha_{j-2,ps}I_{j-2,ps}-\alpha_{j-1,ps}I_{j-1,ps}-\mu_{i\left(j-1\right),ps}I_{j-1,ps}\\ {\overset{•}{I}}_{j,ps}=\alpha_{j-1,ps}I_{j-1,ps}-\gamma_{j,ps}I_{j,ps}-\mu_{ij,ps}I_{j,ps}\\ {\overset{•}{R}}_{j,ps}=\gamma_{j,ps}I_{j,ps}-\mu_{r,ps}R_{ps}. \end{array}$$ (6)

Deer mouse host system:

$$\begin{array}{c} {\overset{•}{S}}_{dm}=\beta S_{dm}-fc_{v}I_{v}\frac{S_{dm}}{N_{dm}}-\mu_{s,dm}S_{dm}\\ {\overset{•}{I}}_{1,dm}=fc_{v}I_{v}\frac{S_{dm}}{N_{dm}}-\alpha_{1,dm}I_{1,dm}-\mu_{i1,dm}I_{1,dm}\\ {\overset{•}{I}}_{2,dm}=\alpha_{1,dm}I_{1,dm}-\alpha_{2,dm}I_{2,dm}-\mu_{i2,dm}I_{2,dm}\\ \vdots \\ {\overset{•}{I}}_{j-1,dm}=\alpha_{j-2,dm}I_{j-2,dm}-\alpha_{j-1,dm}I_{j-1,dm}-\mu_{i\left(j-1\right),dm}I_{j-1,dm}\\ {\overset{•}{I}}_{j,dm}=\alpha_{j-1,dm}I_{j-1,dm}-\gamma_{j,dm}I_{j,dm}-\mu_{ij,dm}I_{j,dm}\\ {\overset{•}{R}}_{j,dm}=\gamma_{j,dm}I_{j,dm}-\mu_{r,dm}R_{dm}. \end{array}$$ (7)

Coupled vector system:

$$\begin{array}{l} {\overset{•}{S}}_{v}=\beta_{v}S_{v}-\frac{fc_{ps}S_{v}}{N_{ps}}\sum_{i=1}^{j}I_{i,ps}-\frac{fc_{dm}S_{v}}{N_{dm}}\sum_{i=1}^{j}I_{i,dm}-\mu_{sv}S_{v}\\ {\overset{•}{I}}_{v}=\frac{fc_{ps}S_{v}}{N_{ps}}\sum_{i=1}^{j}I_{i,ps}+\frac{fc_{dm}S_{v}}{N_{dm}}\sum_{i=1}^{j}I_{i,dm}-\mu_{iv}I_{v}. \end{array}$$ (8)

As with the single host-vector system, we performed equilibrium analysis (S2) and the form of *R*_*0*_ was inferred for *j—1* relapsing rates between *j* infected compartments.

$$R_{0}=f\sqrt{\frac{fc_{v}S_{v}\left(0\right)}{\mu_{iv}}\left[PS+DM\right]}.$$ (9)

Where

$$\begin{array}{c} PS=\frac{c_{ps}}{N_{ps}\left(0\right)}\left[\frac{1}{\left(\propto_{1,ps}+\mu_{i1,ps}\right)}\left[1+\frac{\propto_{1,ps}}{\alpha_{2,ps}+\mu_{i2,ps}}\left[\cdots \left[1+\frac{\propto_{j-1,ps}}{\gamma +\mu_{ij,ps}}\right]\cdots \right]\right]\right]\\ \text{and}\\ DM=\frac{c_{dm}}{N_{dm}\left(0\right)}\left[\frac{1}{\left(\propto_{1,dm}+\mu_{i1,dm}\right)}\left[1+\frac{\propto_{1,dm}}{\alpha_{2,dm}+\mu_{i2,dm}}\left[\cdots \left[1+\frac{\propto_{j-1,dm}}{\gamma +\mu_{ij,dm}}\right]\cdots \right]\right]\right]. \end{array}$$ (10)

**Fig 3:**
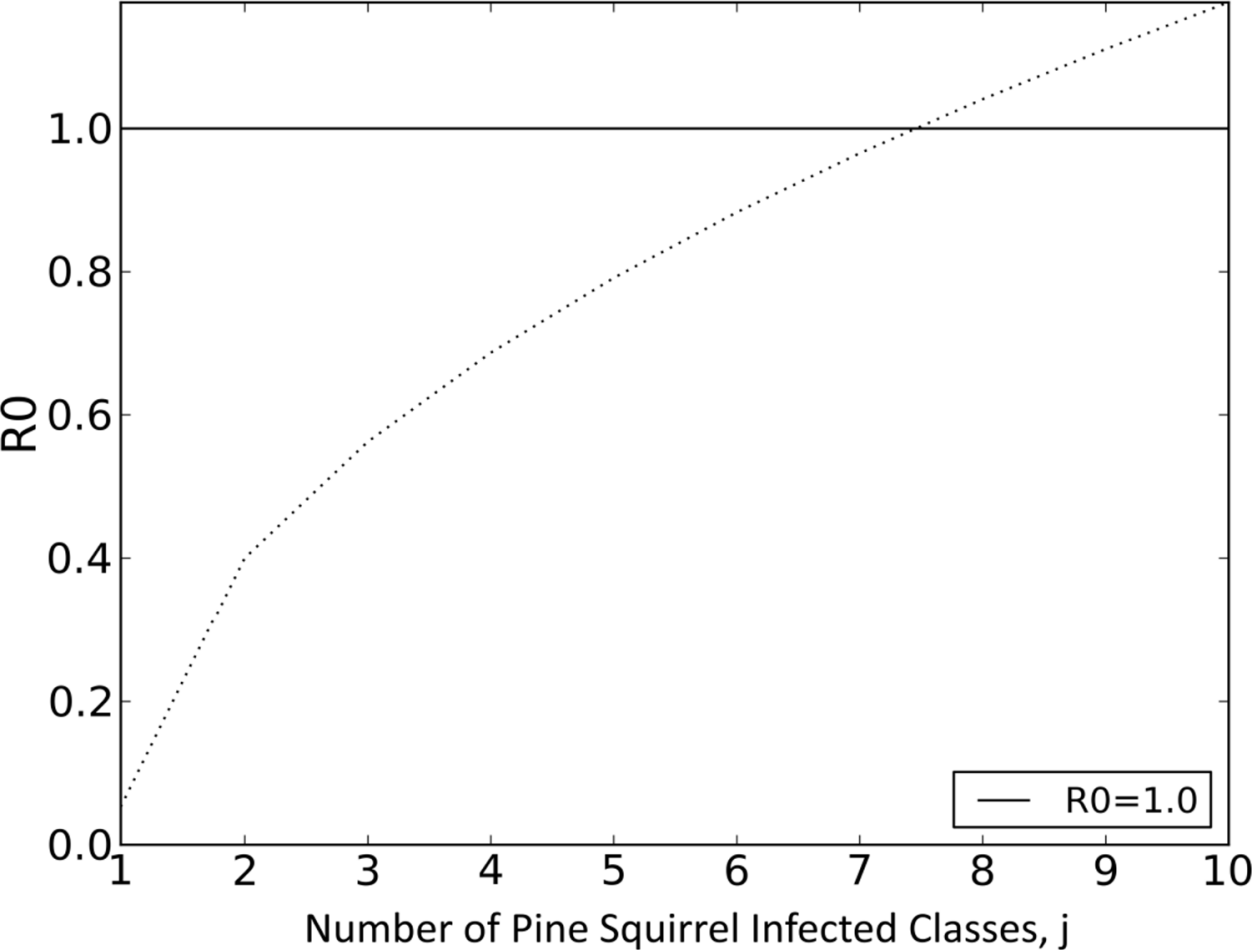
Coupled host-vector system. An incompetent deer mouse (*Peromyscus maniculatus*) host system (*c*_*dm*_ = 0.2) is coupled with a competent pine squirrel (*Tamiasciurus hudsonicus*) host system (*c*_*ps*_ = 0.9). *R*_*0*_ is plotted (Eqs 9 and 10) for the deer mouse host system that contained no relapses and the pine squirrel host system with increasing number of infected compartments. *R*_*0*_ becomes greater than one at seven relapses.

From the coupled host-vector system it is apparent that *R*_*0*_ has the additional dependency for both the host competency values (*c*_*ps*_ and *c*_*dm*_). Since competency values are probabilities between 0 and 1, then they will always decrease the value of *R*_*0*_ as they decrease. Like the single host-vector system, a pattern emerges as more infected compartments are added to each host system (Eqs 9 and 10): a nested sequence of terms that increase the value of *R*_*0*_ and potentially contribute to a change in stability of the DFE. To compare the results of the number of relapses needed for *R*_*0*_ > 1 in the coupled host-vector system with the single host-vector, we added an incompetent deer mouse host system (*c*_*dm*_ = 0.2) and increased the number of relapses in a pine squirrel host system until *R*_*0*_ reached 1. *R*_*0*_ crosses 1 at between *j* = 7 and *j* = 8 infected compartments (seven relapses; Fig 3).

## Discussion

Incorporating a relapsing component into a host-vector SIR modeling framework represents a step towards a better understanding and representation of complex disease systems. We investigated the disease dynamics of TBRF and used the model to better understand the underlying dynamics and interactions among spirochetes, rodent hosts, and tick vectors that contribute to pathogen persistence. Disease models were presented that describes (1) a single host-vector system with a single relapsing class of host individuals, and generalized to *j-1* relapsing host classes and (2) a coupled host-vector model generalized as above to *j -1* relapsing host classes. Analytical techniques allowed for the generalization of *R*_*0*_ with increasing numbers of relapses, and parameters were identified that affect the elimination or persistence of the pathogen (e.g., biting rates, competency values, and population numbers).

In the single host-vector system, *R*_*0*_ is directly proportional to the biting rate (*f*), competency values (*c* and *c*_*v*_), and the ratio of initial vectors to initial hosts $\left(\frac{S_{v}\left(0\right)}{N\left(0\right)}\right).$ An inverse relationship exists between *R*_*0*_ and the vector death rate (*μ*_*iv*_) and the rate that moves individuals out of the infected compartments (α_1,….,_ α_j-1_, *μ*_*i1*_, …, *μ*_*ij*_, and *γ*). When additional relapsing classes are added to the system, *R*_*0*_ always increases because of the addition of a nested sequence of terms that is always > 1 (Eq 5). The coupled host-vector system has similar dependencies with additional interesting dynamics that may be very important to understanding pathogen persistence and host diversity. Coupling of the system with hosts of lower competencies will always reduce *R*_*0*_ (Eqs 9 and 10). As the number of incompetent hosts available as blood meals for infected ticks increases, an effect comparable to the dilution effect occurs and R_0_ always decreases, leading to DFE. The dilution effect states that in the presence of a second, less competent species, competent host-vector encounters leading to transmission events may be replaced by incompetent host-vector encounters that do not end in a pathogen transmission event, thus decreasing *R*_*0*_ [3, 4].

The model presented here addresses the presence of multiple hosts with varying competencies and a single pathogen, however, the model can be extended to address not only differences in host species diversity but also the presence of > 1 pathogen strain. The genetics of *B*. *hermsii* have been well characterized and isolates have been shown to fall into two distinct genomic groups, referred to as genomic group I and II (GGI and GGII) [32, 33]. The presence of both genomic groups of *B*. *hermsii* has been documented on WHI, while only GGII *B*. *hermsii* has been found to date on the mainland around Flathead Lake where host species diversity is greater than that of the WHI.

Field investigations of rodents on WHI confirmed infection in a single deer mouse (*Peromyscus maniculatus*) infected with GGII *B*. *hermsii* (Johnson et al. *In*. *Prep*.). This prompted a laboratory experiment in which we infected deer mice with both GGI and GGII *B*. *hermsii* and monitored them for infection. We challenged deer mice with infection via needle inoculation and infectious tick bite and observed that deer mice show no susceptibility to GGI but are highly susceptible to GGII spirochetes (Johnson et al. *In*. *Prep*.). These findings were in contrast with Burgdorfer and Mavros [16] who were unable to establish infection in deer mice, however, they used infected ticks from a TBRF outbreak near Spokane, WA, U.S.A., which resulted in isolation of GGI *B*. *hermsii*.

The coupled system presented here could be used to examine the effects of not only host species with varying competencies, but also diverse host communities in the presence of *B*. *hermsii* GGI and GGII. The presence of both genomic groups simultaneously may result in a dampening of the dilution effect if GGII is able to infect a diverse array of host species even though GGI is more species limited. Rodent trapping and tick collection on WHI showed one squirrel and one tick infected with GGI and three squirrels infected with GGII. On WHI, 95% of all pine squirrels captured were seropositive for relapsing fever spirochetes while only 4% of deer mice possessed antibodies (Johnson et al. *In Prep*.). All infected individuals at mainland sites with diverse host species were infected with GGII spirochetes (Johnson et al. *In Prep*.).

Although there are limitations to the model presented here, the model is an important first step in understanding a relapsing host-vector disease system. All known complexities of the system were not addressed at this time, including incorporation of GGII strains of *B*. *hermsii* which can infect deer mice and possibly a wide range of other potential hosts (Johnson et al. *In Prep*.). Although there is conflicting evidence at the rate which transovarial transmission of *B*. *hermsii* occurs in *O*. *hermsi*, we can see from the R_0_ calculation that I_v_ does not appear in the equation and therefore will have little impact on disease persistence in the presence of hosts. However, the existence of transovarial transmission may provide insight into the implication of *O*. *hermsi* serving as the reservoir for *B*. *hermsii*, i.e., the ability to maintain infectious ticks in a prolonged absence of competent hosts and/or hosts in general. Additionally, the model could be used to explore drivers in the host and vector communities and prevention/intervention strategies may be explored to identify the effectiveness of host control versus vector control. Further, this may provide insight into human protective measures and the effectiveness of control strategies such as host vaccination; simulations could be run to assess the efficacy of control programs such as vaccination regimes and vector control.

Ecological factors including biotic and abiotic interactions may play a primary role in the emergence and persistence of infectious diseases [34–39]. Understanding the complete epidemiology of a disease is crucial to advancing the ability to predict and control outbreaks in human and wildlife populations, however, this is rarely an attainable goal. Sonenshine [40] outlines the sequence of steps typically undertaken when attempting to understand the epidemiology of a given system. The pathway typically begins with the identification of a clinical syndrome, followed by discovery of the causative disease agent, and then the identification of the source of the agent in nature. The final step includes investigating the often complex biology and ecology of the hosts and/or vectors involved. Given the difficulty frequently encountered when attempting to study a disease in nature, the last step is often the most difficult. The application of advanced modeling techniques to poorly understood systems is often the only way to begin to understand the drivers of these systems.

The ecological dynamics of relapsing fever systems around the world are poorly understood. Here we use a North American system of relapsing fever caused by *B*. *hermsii*; however, information gathered from this modeling exercise can be applied to TBRF systems around the world. TBRF remains a major public health threat in Africa [41]. In addition to other TBRF systems, the ideas presented here may provide the groundwork for relapsing components to be included in other disease systems with greater public health implications such as malaria.

## Supporting Information

S1 Appendix Single host-vector system: Equilibrium analysis. (DOCX)

S2 Appendix Coupled host-vector system: Equilibrium analysis. (DOCX)
