# Supplementary material for: Modeling Relapsing Disease Dynamics in a Host-Vector Community
Source: PLoS Negl Trop Dis. 2016 Feb 24;10(2):e0004428. doi: 10.1371/journal.pntd.0004428 (PMC4765964; doi:10.1371/journal.pntd.0004428)
Supplement: S1 Appendix — (DOCX) [file pntd.0004428.s001.docx]

**S1 Appendix**

**Single Host-vector System – Equilibrium Analysis**

The generalized system for the infection dynamics in a single host-vector system with *j* - 1 relapsing rates for *j =* 1 infected compartments describes the number of susceptible hosts *S*(t), infectious hosts *I_k_*(t), removed hosts *R*(t), susceptible vectors *S_v_*(t), and infected vectors *I_v_*(t), where the total host population is $N=S+\sum_{k=1}^{j} I_{k}+R$ and the total vector population is *N_v_* = *S_v_* + *I_v_* (see Fig 1a for a compartmental diagram and Table 1 for parameter definitions). The equations are

Host equations: (A1.1)

$$\begin{matrix} \mathop{S}\limits^{\bullet} =\beta S-fc_{v}I_{v}\frac{S}{N}-\mu_{s}S \\ \mathop{I_{1}}\limits^{\bullet} =fc_{v}I_{v}\frac{S}{N}-\alpha_{1}I_{1}-\mu_{i1}I_{1} \\ \mathop{I_{2}}\limits^{\bullet} =\alpha_{1}I_{1}-\alpha_{2}I_{2}-\mu_{i2}I_{2} \end{matrix}$$

**.**

**.**

**.**

Vector equations: (A1.2)

$$\begin{matrix} \mathop{S_{v}}\limits^{\bullet} =\beta_{v}S_{v}-\frac{fcS_{v}}{N}\sum_{i=1}^{j} I_{i}-\mu_{sv}S_{v} \\ \mathop{I_{v}}\limits^{\bullet} =\frac{fcS_{v}}{N}\sum_{i=1}^{j} I_{i}-\mu_{iv}I_{v}. \end{matrix}$$

In the absence of disease, the system steady state is (*S*^*^, *I^*^_1_*, …, *I^*^_j_*, *R^*^*, *S^*^_v_*, *I^*^_v_*) = (*S*(0), 0, ..., 0, *S_v_*(0),0) for *j* infected compartments, where now the total populations become *N* = *S*(0) and *N_v_* = *S_v_*(0). This steady state is defined to be the disease free equilibrium (DFE). An equilibrium stability analysis is performed on the DFE by the extraction of *R_0_*. A single host-vector model with no relapses (*j* = 1) is first analyzed. The variables in this model are the number of susceptible hosts *S*(t), infectious hosts *I_1_*(t), removed hosts *R*(t), susceptible vectors *S_v_*(t), and infected vectors *I_v_*(t). The total host population is *N = S + I_1_* + *R*, and the total vector population is *N_v_* = *S_v_* + *I_v_*. The mathematics is simplified by considering an equivalent non-dimensional system. First a new time scale is defined,  = γt, and the equations are scaled by the initial host population, *N*(0). The resulting rescaled host variables, *s*, *i_1_*, and *r*, indicate the fraction of the initial population in the susceptible, infectious, and recovered classes, and the total non-dimensional host population becomes *n* = *s + i_1_* + *r*. The vector compartments, *s_v_* and *i_v_*, represent susceptible and infectious vectors, respectively, normalized by *N*(0). The total vector population thus becomes *n_v_* = *s_v_* + *i_v_*. The resulting dimensionless system is

Host equations: (A1.3)

$$\begin{matrix} \frac{ds}{d\tau}=an-ki_{v}\frac{s}{n}-b_{s}s \\ \frac{di_{1}}{d\tau}=ki_{v}\frac{s}{n}i_{1}-i_{1}-b_{i1}i_{1} \\ \frac{dr}{d\tau}=i_{1}-b_{r}r. \end{matrix}$$

Vector equations: (A1.4)

$$\begin{matrix} \frac{ds_{v}}{d\tau}=a_{v}s_{v}-ls_{v}\frac{i_{1}}{n}-b_{sv}s_{v} \\ \frac{di_{v}}{d\tau}=ls_{v}\frac{i_{1}}{n}-b_{iv}i_{v}, \end{matrix}$$

where parameters are as shown in Table 1.

To evaluate the invasiveness of the disease in this system, we extract *R_0_* following the techniques developed by van den Driessche and Watmough (2002). The size of the system is first reduced by considering only *i_1_* and *i_v_*, and the rate of appearance of new infections and the rate of transfer between compartments for all other processes respectively,

$\frac{d}{d\tau}\left[\begin{matrix} i_{1} \\ i_{v} \end{matrix}\right]=w-v=\left[\begin{matrix} ki_{v}\frac{s}{n} \\ ls_{v}\frac{i_{1}}{n} \end{matrix}\right]-\left[\begin{matrix} i_{1}+b_{i1}i_{1} \\ b_{iv}i_{v} \end{matrix}\right].$ (A1.5)

The Jacobians of the vector fields *w* and *v* evaluated at the DFE describe the linearization about the DFE in terms of new infection occurrences and are


$W\left(1,0,0,\frac{S_{v}\left(0\right)}{N\left(0\right)},0\right)=\left[\begin{matrix} 0 & k \\ l\frac{S_{v}\left(0\right)}{N\left(0\right)} & 0 \end{matrix}\right]andV\left(1,0,0,\frac{S_{v}\left(0\right)}{N\left(0\right)},0\right)=\left[\begin{matrix} 1+b_{i1} & 0 \\ 0 & b_{iv} \end{matrix}\right].$ (A1.6)

The basic reproductive number, *R_0_*, is given by the dominant eigenvalue of *WV^-1^* (see Theorem 2 from van den Driessche and Watmough 2002), which is written in terms of the original variables and parameters as

$R_{0}=f\sqrt{\frac{cc_{v}}{\mu_{iv}}\frac{S_{v}\left(0\right)}{N\left(0\right)}\left[\frac{1}{\gamma+\mu_{i1}}\right]}.$ (A1.7)

For the system with one relapse (*j* = 2 infected compartments), the host compartments, *s*, *i_1_*, *i_2_*, and *r*, indicate the fractions of the initial population in the susceptible, infectious, and recovered classes, so that the total rescaled host population is *n = s + i_1_* + *i_2_* + *r*. The vector compartments, *s_v_* and *i_v_*, represent susceptible and infectious vectors, respectively, scaled to the initial number of hosts. The total vector population thus becomes *n_v_* = *s_v_* + *i_v_*. The resulting dimensionless system is

Host equations: (A1.8)

$$\begin{matrix} \frac{ds}{d\tau}=an-ki_{v}\frac{s}{n}-b_{s}s \\ \frac{di_{1}}{d\tau}=ki_{v}\frac{s}{n}i_{1}-q_{1}i_{1}-b_{i1}i_{1} \\ \frac{di_{2}}{d\tau}=q_{1}i_{1}-i_{2}-b_{i2}i_{2} \\ \frac{dr}{d\tau}=i_{2}-b_{r}r. \end{matrix}$$

Vector equations: (A1.9)

$$\begin{matrix} \frac{ds_{v}}{d\tau}=a_{v}s_{v}-ls_{v}\frac{i_{1}}{n}-ls_{v}\frac{i_{2}}{n}-b_{sv}s_{v} \\ \frac{di_{v}}{d\tau}=ls_{v}\frac{i_{1}}{n}+ls_{v}\frac{i_{2}}{n}-b_{iv}i_{v}. \end{matrix}$$

Computing *R_0_* the reduced set of equations become

$\frac{d}{d\tau}\left[\begin{matrix} i_{1} \\ i_{2} \\ i_{v} \end{matrix}\right]=w-v=\left[\begin{matrix} ki_{v}\frac{s}{n} \\ 0 \\ ls_{v}\frac{i_{1}}{n}+ls_{v}\frac{i_{2}}{n} \end{matrix}\right]-\left[\begin{matrix} q_{1}i_{1}+b_{i1}i_{1} \\ -q_{1}i_{1}+i_{2}+b_{i2}i_{2} \\ b_{iv}i_{v} \end{matrix}\right].$ (A1.10)

Note that only progression from *s* to *i_1_* and *s_v_* to *i_v_* are considered to be new infections. The corresponding Jacobian matrices of *w* and *v* evaluated at the DFE are

$W=\left[\begin{matrix} 0 & 0 & k \\ 0 & 0 & 0 \\ l\frac{S_{v}\left(0\right)}{N\left(0\right)} & l\frac{S_{v}\left(0\right)}{N\left(0\right)} & 0 \end{matrix}\right]and V=\left[\begin{matrix} q_{1}+b_{i1} & 0 & 0 \\ -q_{1} & 1+b_{i2} & 0 \\ 0 & 0 & b_{iv} \end{matrix}\right]$ (A1.11)

and the dominant eigenvalue of *WV^-1^*, which, in terms of the original variables and parameters is

$R_{0}=f\sqrt{\frac{cc_{v}}{\mu_{iv}}\frac{S_{v}\left(0\right)}{N\left(0\right)}\left[\frac{1}{\alpha_{1}+\mu_{i1}}\left[1+\frac{\alpha_{1}}{\gamma+\mu_{i2}}\right]\right]}.$ (A1.12)

The form of *R_0_* can now be inferred for *j - 1* relapsing rates between *j* infected compartments as

$R_{0}=f\sqrt{\frac{cc_{v}}{\mu_{iv}}\frac{S_{v}\left(0\right)}{N\left(0\right)}\left[\frac{1}{\alpha_{1}+\mu_{i1}}\left[1+\frac{\alpha_{1}}{\alpha_{2}+\mu_{i2}}\left[\cdots\left[1+\frac{\alpha_{j-1}}{\gamma+\mu_{ij}}\right]\right]\right]\right]}.$ (A1.13)
