# Supplementary material for: Modeling Relapsing Disease Dynamics in a Host-Vector Community
Source: PLoS Negl Trop Dis. 2016 Feb 24;10(2):e0004428. doi: 10.1371/journal.pntd.0004428 (PMC4765964; doi:10.1371/journal.pntd.0004428)
Supplement: S2 Appendix — (DOCX) [file pntd.0004428.s002.docx]

**S2 Appendix**

**Coupled Host-vector System – Equilibrium Analysis**

The generalized system for the infection dynamics in a coupled host-vector system with *j* - 1 relapsing rates for *j =* 1 infected compartments describes the pine squirrel system with the number of susceptible hosts *S_ps_*(t), infectious hosts *I_k,ps_*(t), and removed hosts *R_ps_*(t). The total pine squirrel host population is Likewise, the deer mouse host system consists of susceptible hosts *S_dm_*(t), infectious hosts *I_k,dm_*(t), and removed hosts *R_dm_*(t) with a total deer mouse host population of The vector compartments are susceptible vectors *S_v_*(t), infected vectors *I_v_*(t) and a total vector population of *N_v_* = *S_v_* + *I_v_*. The equations are

Pine squirrel host system: (S2.1)

$$\begin{matrix} \mathop{S_{ps}}\limits^{\bullet} =\beta S_{ps}-fc_{v}I_{v}\frac{S_{ps}}{N_{ps}}-\mu_{s,ps}S_{ps} \\ \mathop{I_{1,ps}}\limits^{\bullet} =fc_{v}I_{v}\frac{S_{ps}}{N_{ps}}-\alpha_{1,ps}I_{1,ps}-\mu_{i1,ps}I_{1,ps} \\ \mathop{I_{2,ps}}\limits^{\bullet} =\alpha_{1,ps}I_{1,ps}-\alpha_{2,ps}I_{2,ps}-\mu_{i2,ps}I_{2,ps} \\ \vdots\\ \mathop{I_{j-1,ps}}\limits^{\bullet} =\alpha_{j-2,ps}I_{j-2,ps}-\alpha_{j-1,ps}I_{j-1,ps}-\mu_{i\left(j-1\right),ps}I_{j-1,ps} \\ \mathop{I_{j,ps}}\limits^{\bullet} =\alpha_{j-1,ps}I_{j-1,ps}-\gamma_{j,ps}I_{j,ps}-\mu_{ij,ps}I_{j,ps} \\ \mathop{R_{j,ps}}\limits^{\bullet} =\gamma_{j,ps}I_{j,ps}-\mu_{r,ps}R_{ps}. \end{matrix}$$

Deer mouse host system: (S2.2)

$\begin{matrix} \mathop{S_{dm}}\limits^{\bullet} =\beta S_{dm}-fc_{v}I_{v}\frac{S_{dm}}{N_{dm}}-\mu_{s,dm}S_{dm} \\ \mathop{I_{1,dm}}\limits^{\bullet} =fc_{v}I_{v}\frac{S_{dm}}{N_{dm}}-\alpha_{1,dm}I_{1,dm}-\mu_{i1,dm}I_{1,dm} \\ \mathop{I_{2,dm}}\limits^{\bullet} =\alpha_{1,dm}I_{1,dm}-\alpha_{2,dm}I_{2,dm}-\mu_{i2,dm}I_{2,dm} \\ \vdots\\ \mathop{I_{j-1,dm}}\limits^{\bullet} =\alpha_{j-2,dm}I_{j-2,dm}-\alpha_{j-1,dm}I_{j-1,dm}-\mu_{i\left(j-1\right),dm}I_{j-1,dm} \\ \mathop{I_{j,dm}}\limits^{\bullet} =\alpha_{j-1,dm}I_{j-1,dm}-\gamma_{j,dm}I_{j,dm}-\mu_{ij,dm}I_{j,dm} \\ \mathop{R_{j,dm}}\limits^{\bullet} =\gamma_{j,dm}I_{j,dm}-\mu_{r,dm}R_{dm}. \end{matrix}$

Coupled vector system: (S2.3)

$$\begin{matrix} \mathop{S_{v}}\limits^{\bullet} =\beta_{v}S_{v}-\frac{fc_{ps}S_{v}}{N_{ps}}\sum_{i=1}^{j} I_{i,ps}-\frac{fc_{dm}S_{v}}{N_{dm}}\sum_{i=1}^{j} I_{i,dm}-\mu_{sv}S_{v} \\ \mathop{I_{v}}\limits^{\bullet} =\frac{fc_{ps}S_{v}}{N_{ps}}\sum_{i=1}^{j} I_{i,ps}+\frac{fc_{dm}S_{v}}{N_{dm}}\sum_{i=1}^{j} I_{i,dm}-\mu_{iv}I_{v}. \end{matrix}$$

**Equilibrium analysis**

To understand the effect of an additional host system, a coupled host-vector model with no relapses is first analyzed following the techniques from the single host-vector system. The resulting dimensionless system is

Pine squirrel host system: (S2.4)

$$\begin{matrix} \frac{ds_{ps}}{d\tau}=a_{ps}s_{ps}-ki_{v}\frac{s_{ps}}{n_{ps}}-b_{s,ps}s_{ps} \\ \frac{di_{1,ps}}{d\tau}=ki_{v}\frac{s_{ps}}{n_{ps}}-i_{1,ps}-b_{i1,ps}i_{1,ps} \\ \frac{dr_{ps}}{d\tau}=i_{1,ps}-b_{r,ps}r_{ps}. \end{matrix}$$

Deer mouse host system: (S2.5)

$$\begin{matrix} \frac{ds_{dm}}{d\tau}=a_{dm}s_{dm}-ki_{v}\frac{s_{dm}}{n_{dm}}-b_{s,dm}s_{dm} \\ \frac{di_{1,dm}}{d\tau}=ki_{v}\frac{s_{dm}}{n_{dm}}-i_{1,dm}-b_{i1,dm}i_{1,dm} \\ \frac{dr_{dm}}{d\tau}=i_{1,dm}-b_{r,dm}r_{dm}. \end{matrix}$$

Coupled vector system: (S2.6)

$$\begin{matrix} \frac{ds_{v}}{d\tau}=a_{v}s_{v}-l_{rs}s_{v}\frac{i_{1,ps}}{n_{ps}}-l_{dm}s_{v}\frac{i_{1,dm}}{n_{dm}}-b_{sv}s_{v} \\ \frac{di_{v}}{d\tau}=l_{ps}s_{v}\frac{i_{1,ps}}{n_{ps}}+l_{dm}s_{v}\frac{i_{1,dm}}{n_{dm}}-b_{iv}i. \end{matrix}$$

where parameters are as shown in Table 1.

In the absence of disease, the system steady state is$S_{ps}^{*},I_{1,ps}^{*},R_{ps}^{*},S_{v,ps}^{*},I_{v,ps}^{*},S_{dm}^{*},I_{1,dm}^{*},R_{dm}^{*})=\left(S_{ps}\left(0\right),0,0,s_{v}\left(0\right),S_{dm},\left(0\right),0,0\right).$ The total population becomes$N_{ps}=S_{ps}\left(0\right),N_{dm}=S_{dm}\left(0\right),N_{v}=S_{v}\left(0\right).$ This steady state is the DFE for the coupled host-vector system with no relapses.

The size of the system is reduced by considering only *i_1,ps_, i_v_,* and *i_1,dm_* and the rate of appearance of new infections and the rate of transfer between compartments for all other processes respectively,

$\frac{d}{d\tau}\left[\begin{matrix} i_{1,ps} \\ i_{v} \\ i_{2,dm} \end{matrix}\right]=w-v=\left[\begin{matrix} ki_{v}\frac{s_{ps}}{n_{ps}} \\ l_{ps}s_{v}\frac{i_{1,ps}}{n_{ps}}+l_{dm}s_{v}\frac{i_{1,dm}}{n_{dm}} \\ ki_{v}\frac{s_{dm}}{n_{dm}} \end{matrix}\right]-\left[\begin{matrix} i_{1,ps}+b_{i1,ps}i_{1,ps} \\ b_{iv}i_{v} \\ i_{1,dm}+b_{i1,dm}i_{1,dm} \end{matrix}\right].$ (S2.7)

The Jacobians of the vector fields *w* and *v* evaluated at the DFE are

$W=\left[\begin{matrix} 0 & k & 0 \\ l_{ps}\frac{S_{v}\left(0\right)}{N\left(0\right)} & 0 & l_{dm}\frac{S_{v}\left(0\right)}{N\left(0\right)} \\ 0 & k & 0 \end{matrix}\right],\text{and }V=\left[\begin{matrix} 1+b_{i1,ps} & 0 & 0 \\ 0 & b_{iv} & 0 \\ 0 & 0 & 1+b_{i1,dm} \end{matrix}\right]$ (S2.8)

and the dominant eigenvalue of *WV^-1^* written in terms of the original variables and parameters is

$R_{0}=f\sqrt{\frac{fc_{v}S_{v}\left(0\right)}{\mu_{iv}} \left[\frac{c_{ps}}{N_{ps}\left(0\right)}\left[\frac{1}{\left(\gamma+\mu_{i1,ps}\right)}\right]+\frac{c_{dm}}{N_{dm}\left(0\right)}\left[\frac{1}{\left(\gamma+\mu_{i1,dm}\right)}\right]\right].}$ (S2.9)

For the system with one relapse (*j* = 2 infected compartments), the host compartments, *s*, *i_1_*, *i_2_*, and *r*, indicate the fractions of the initial population in the susceptible, infectious, and recovered classes, so that the total rescaled host population is *n = s + i_1_* + *i_2_* + *r*. The vector compartments, *s_v_* and *i_v_*, represent susceptible and infectious vectors, respectively, scaled to the initial number of hosts. The total vector population thus becomes *n_v_* = *s_v_* + *i_v_*. The resulting dimensionless system is

Pine squirrel host system: (S2.10)

$$\begin{matrix} \frac{ds_{ps}}{d\tau}=a_{ps}s_{ps}-ki_{v}\frac{s_{ps}}{n_{ps}}-b_{s,ps}s_{ps} \\ \frac{di_{1,ps}}{d\tau}=ki_{v}\frac{s_{ps}}{n_{ps}}-q_{1,ps}i_{1,ps}-b_{i1,ps}i_{1,ps} \\ \frac{di_{2,ps}}{d\tau}=q_{1,ps}i_{1,ps}-i_{2,ps}-b_{i2,ps}i_{2,ps} \\ \frac{dr_{ps}}{d\tau}=i_{1,ps}-b_{r,ps}r_{ps}. \end{matrix}$$

Deer mouse host system: (S2.11)

$$\begin{matrix} \frac{ds_{dm}}{d\tau}=a_{dm}s_{dm}-ki_{v}\frac{s_{dm}}{n_{dm}}-b_{s,dm}s_{dm} \\ \frac{di_{1,dm}}{d\tau}=ki_{v}\frac{s_{dm}}{n_{dm}}-i_{1,dm}-b_{i1,dm}i_{1,dm} \\ \frac{di_{2,dm}}{d\tau}=q_{1,dm}i_{1,dm}-i_{2,dm}-b_{i2,dm}i_{2,dm} \\ \frac{dr_{dm}}{d\tau}=i_{1,dm}-b_{r,dm}r_{dm}. \end{matrix}$$

Coupled vector system: (S2.12)

$$\begin{matrix} \frac{ds_{v}}{d\tau}=a_{v}s_{v}-l_{rs}s_{v}\frac{i_{1,ps}}{n_{ps}}-l_{ps}s_{v}\frac{i_{2,ps}}{n_{ps}}-l_{dm}s_{v}\frac{i_{1,dm}}{n_{dm}}-l_{dm}s_{v}\frac{i_{2,dm}}{n_{dm}}-b_{sv}s_{v} \\ \frac{di_{v}}{d\tau}=l_{ps}s_{v}\frac{i_{1,ps}}{n_{ps}}+l_{ps}s_{v}\frac{i_{2,ps}}{n_{ps}}+l_{dm}s_{v}\frac{i_{1,dm}}{n_{dm}}+l_{dm}s_{v}\frac{i_{2,dm}}{n_{dm}}-b_{iv}i. \end{matrix}$$

For computing *R_0_* the reduced set of equations are

$\frac{d}{d\tau}\left[\begin{matrix} i_{1,ps} \\ i_{2,ps} \\ i_{v} \\ i_{1,dm} \\ i_{2,dm} \end{matrix}\right]=w-v=\left[\begin{matrix} ki_{v}\frac{s_{ps}}{n_{ps}} \\ 0 \\ l_{ps}s_{v}\frac{i_{1,ps}}{n_{ps}}+l_{dm}s_{v}\frac{i_{1,dm}}{n_{dm}} \\ 0 \\ ki_{v}\frac{s_{dm}}{n_{dm}} \end{matrix}\right]-\left[\begin{matrix} q_{1,ps}i_{1,ps}+b_{i1,ps}i_{1,ps} \\ -q_{1,ps}i_{1,ps}+i_{2,ps}+b_{i2,ps}i_{2,ps} \\ b_{iv}i_{v} \\ q_{1,dm}i_{1,dm}+b_{i1,dm}i_{1,dm} \\ -q_{1,dm}i_{1,dm}+i_{2,dm}+b_{i2,dm}i_{2,dm} \end{matrix}\right].$ (S2.13)

The Jacobians of the vector fields *w* and *v* evaluated at the DFE are

$\begin{matrix} W=\left[\begin{matrix} 0 & 0 & k & 0 & 0 \\ 0 & 0 & 0 & 0 & 0 \\ l_{ps}\frac{S_{v}\left(0\right)}{N\left(0\right)} & l_{ps}\frac{S_{v}\left(0\right)}{N\left(0\right)} & 0 & l_{dm}\frac{S_{v}\left(0\right)}{N\left(0\right)} & l_{dm}\frac{S_{v}\left(0\right)}{N\left(0\right)} \\ 0 & 0 & 0 & 0 & 0 \\ 0 & 0 & k & 0 & 0 \end{matrix}\right] \\ \text{and} \\ V=\left[\begin{matrix} q_{1,ps}+b_{i1,ps} & 0 & 0 & 0 & 0 \\ -q_{1,ps} & 1+b_{i2,ps} & 0 & 0 & 0 \\ 0 & 0 & b_{iv} & 0 & 0 \\ 0 & 0 & 0 & q_{1,dm}+b_{i1,dm} & 0 \\ 0 & 0 & 0 & -q_{1,dm} & 1+b_{i2,dm} \end{matrix}\right] \end{matrix}$ (S2.14)

and the dominant eigenvalue of *WV^-1^* written in terms of the original variables and parameters is $R_{0}=f\sqrt{\frac{fc_{v}S_{v}\left(0\right)}{\mu_{iv}}\left[\frac{c_{ps}}{N_{ps}\left(0\right)}\left[\frac{1}{\left(\alpha_{1,ps}+\mu_{i1,ps}\right)}\left[1+\frac{\alpha_{1,ps}}{\gamma+\mu_{i2,ps}}\right]\right]+\frac{c_{dm}}{N_{_{dm}}\left(0\right)}\left[\frac{1}{\alpha_{1,dm}+\mu_{i1,dm}}\left[1+\frac{\alpha_{1,dm}}{\gamma+\mu_{i2,dm}}\right]\right]\right]}.(30)$

The form of *R_0_* can now be inferred for *j - 1* relapsing rates between *j* infected compartments.

$R_{0}=f\sqrt{\frac{fc_{v}S_{v}\left(0\right)}{\mu_{iv}} \left[PS+DM\right]}.$ (S2.16)

Where

$\begin{matrix} PS=\frac{c_{ps}}{N_{ps}\left(0\right)}\left[\frac{1}{\left(\propto_{1,ps}+\mu_{i1,ps}\right)}\left[1+\frac{\propto_{1,ps}}{\alpha_{2,ps}+\mu_{i2,ps}}\left[\cdots\left[1+\frac{\propto_{j-1,ps}}{\gamma+\mu_{ij,ps}}\right]\cdots\right]\right]\right] \\ \text{and} \\ DM=\frac{c_{dm}}{N_{dm}\left(0\right)}\left[\frac{1}{\left(\propto_{1,dm}+\mu_{i1,dm}\right)}\left[1+\frac{\propto_{1,dm}}{\alpha_{2,dm}+\mu_{i2,dm}}\left[\cdots\left[1+\frac{\propto_{j-1,dm}}{\gamma+\mu_{ij,dm}}\right]\cdots\right]\right]\right]. \end{matrix}$ (S2.17)
